# Peptide hormone ELABELA enhances extravillous trophoblast differentiation, but placenta is not the major source of circulating ELABELA in pregnancy

**DOI:** 10.1038/s41598-019-55650-5

**Published:** 2019-12-13

**Authors:** Danai Georgiadou, Souad Boussata, Willemijn H. M. Ranzijn, Leah E. A. Root, Sanne Hillenius, Jeske M. bij de Weg, Carolien N. H. Abheiden, Marjon A. de Boer, Johanna I. P. de Vries, Tanja G. M. Vrijkotte, Cornelis B. Lambalk, Esther A. M. Kuijper, Gijs B. Afink, Marie van Dijk

**Affiliations:** 1 Reproductive Biology Laboratory, Amsterdam University Medical Centers, location AMC, Reproduction & Development Research Institute, Amsterdam, The Netherlands; 2 Department of Obstetrics & Gynaecology, Amsterdam University Medical Centers, location VUmc, Reproduction & Development Research Institute, Amsterdam, The Netherlands; 3 Department of Public Health, Amsterdam University Medical Centers, location AMC, Amsterdam, The Netherlands; 4 Reproductive Medicine, Department of Obstetrics & Gynaecology, Amsterdam University Medical Centers, location VUmc, Amsterdam Reproduction & Development Research Institute, Amsterdam, The Netherlands

**Keywords:** Differentiation, Endocrinology, Predictive markers, Hypertension

## Abstract

Preeclampsia is a frequent gestational hypertensive disorder with equivocal pathophysiology. Knockout of peptide hormone ELABELA (ELA) has been shown to cause preeclampsia-like symptoms in mice. However, the role of ELA in human placentation and whether ELA is involved in the development of preeclampsia in humans is not yet known. In this study, we show that exogenous administration of ELA peptide is able to increase invasiveness of extravillous trophoblasts *in vitro*, is able to change outgrowth morphology and reduce trophoblast proliferation *ex vivo*, and that these effects are, at least in part, independent of signaling through the Apelin Receptor (APLNR). Moreover, we show that circulating levels of ELA are highly variable between women, correlate with BMI, but are significantly reduced in first trimester plasma of women with a healthy BMI later developing preeclampsia. We conclude that the large variability and BMI dependence of ELA levels in circulation make this peptide an unlikely candidate to function as a first trimester preeclampsia screening biomarker, while in the future administering ELA or a derivative might be considered as a potential preeclampsia treatment option as ELA is able to drive extravillous trophoblast differentiation.

## Introduction

The placenta orchestrates the normal growth and development of the fetus while serving as an interface with the maternal environment. Protagonists of the process of placentation are highly invasive placental cells, known as extravillous trophoblasts. These cells originate from villous cytotrophoblasts and are able to undergo epithelial mesenchymal transition in which they differentiate from a proliferative to an invasive phenotype. The latter cell type is responsible for remodeling of the maternal spiral arteries by infiltrating into the decidua and myometrium during the initial steps of placentation taking place in the first trimester of pregnancy. In this way, they establish a proper connection between the mother and the fetus. Thus, any placental cellular dysfunction potentially leads to gestational complications such as hypertensive disorders of pregnancy (pregnancy induced hypertension, preeclampsia, eclampsia and HELLP) and fetal growth restriction^1^. Preeclampsia (PE) has an average incidence of up to 10% in human pregnancies worldwide and contributes to maternal mortality, morbidity as well as perinatal morbidity such as small for gestational age infants and prematurity^2–4^. The clinical profile of preeclampsia consists of the presence of *de novo* hypertension after 20 weeks’ gestation combined with proteinuria and/or maternal acute kidney injury, liver dysfunction, neurological features, haemolysis or thrombocytopenia, and can overlap and occur with fetal growth restriction^5^. Although the clinical profile of preeclampsia is quite distinct, the underlying mechanism of its pathophysiology still remains unclear. Additionally, there is no known treatment other than terminating the pregnancy and delivering of the placenta which, especially in cases of early-onset PE with symptoms appearing before 34 weeks of gestation^6,7^, can have detrimental effects for the premature neonate. Knowledge on reducing the risk for preeclampsia is modest as well the implementation of this knowledge^4,8,9^. Therefore, identification of disease-causing factors, investigation of their normal function in placentation and the prospective development of reliable biomarkers that allow early diagnosis are considered vital.

ELABELA (APELA/ELA/Toddler) is an endogenous secreted hormonal peptide of 32 amino acids that signals via the Apelin Receptor (APLNR or APJ). ELA has restricted expression in adult human tissues where its transcripts have been found in endothelium, kidney and prostate^10^. It is highly secreted by human embryonic stem cells (hESCs) and plays a key role in cardiovascular development, embryonic cell self-renewal and apoptosis, which indicates its critical contribution during embryonic development^11^. Interestingly, the so far only known receptor of ELA, APLNR, is not present in hESCs, implicating that ELA acts through a secondary receptor that appears to be located at the cell surface and signals through the PI3K/AKT signaling pathway^12^. This second receptor remains thus far unidentified. Knockout of ELA in pregnant mice decreases the vascularization of the placental labyrinth, due to increased cell death and a low differentiation rate of syncytiotrophoblasts^13^. Therefore, these effects link ELA with angiogenesis, and subsequently with the regulation of placentation and embryo development in rodents. In addition, pregnant ELA knockout mice develop preeclampsia-like symptoms, i.e. high blood pressure and proteinuria, symptoms that were rescued after the exogenous administration of ELA^13^.

As both ELA and Apelin have been shown to be implicated in preeclampsia pathophysiology in rodents^13,14^, translation of this to humans is necessary. We have previously shown that ELA is expressed in human first trimester placenta and is able to increase invasion of trophoblast-like JAR choriocarcinoma cells^13^. Based on these data, we hypothesized that ELA plays a role in controlling aspects of extravillous trophoblast biology. In this study, we investigated the role of the hormone ELA in the regulation of differentiation of human extravillous trophoblasts from a proliferative to invasive phenotype. By employing the HTR8/SVneo extravillous trophoblast cell line and human first trimester placental explants we found that ELA enhances extravillous trophoblast differentiation but that these effects are, at least in part, independent of signaling through APLNR. Furthermore, we investigate the expression of ELA in healthy and preeclamptic placental tissue, and its circulating levels in three different cohorts of healthy and preeclamptic pregnancies. Using these cohorts we show that circulating levels of ELA are highly variable between women, correlate with BMI, but appear to be significantly reduced in first trimester plasma of women with a healthy BMI later developing preeclampsia.

## Results

### ELA and Apelin are able to increase the invasion capacity of HTR8/SVneo cells

**Figure 1:**
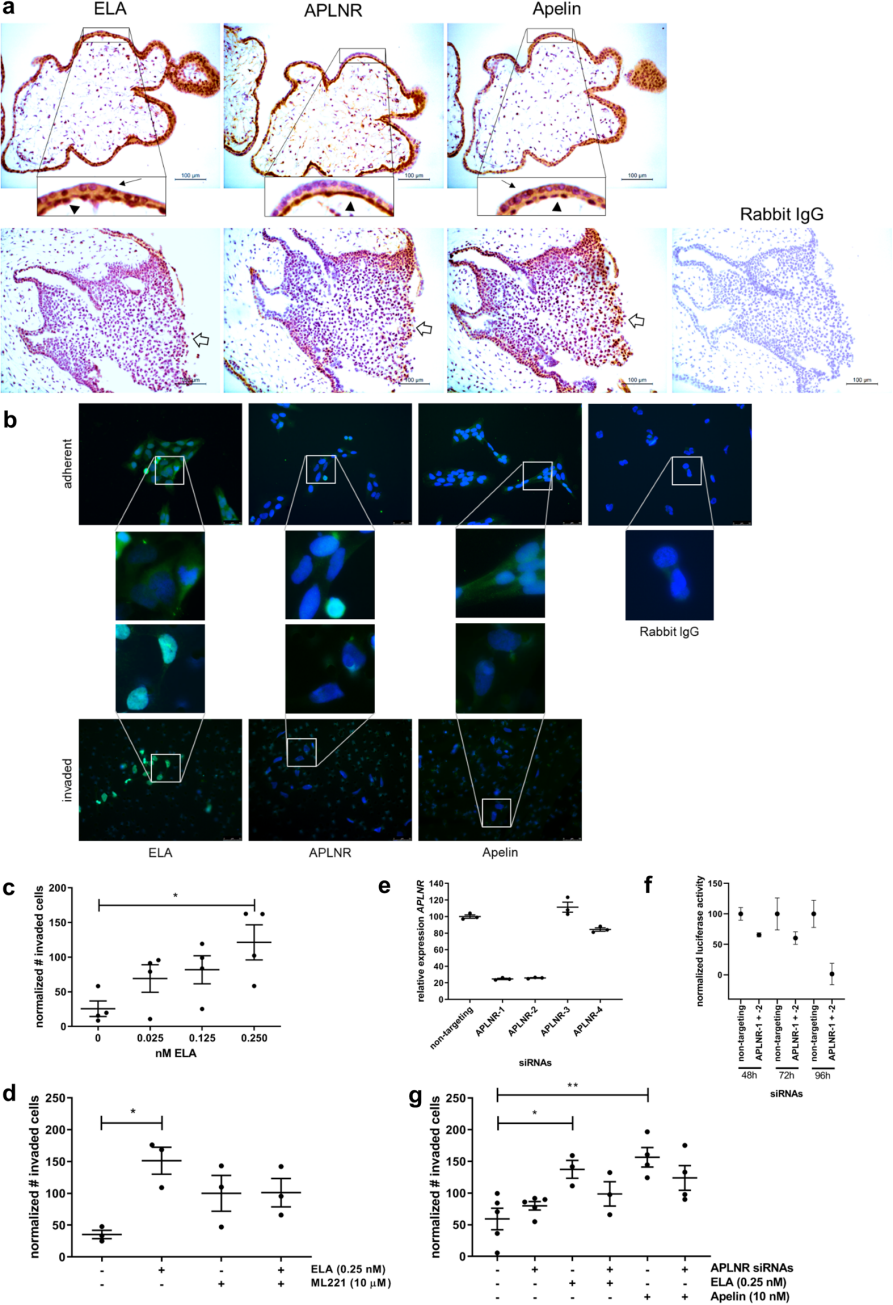
ELA and Apelin increase invasion capacity of HTR8/SVneo cells. (**a**) Immunohistochemistry shows ELA, APLNR and Apelin to be expressed in first trimester human placenta villous cytotrophoblasts (arrowheads) and distal column extravillous trophoblasts (open arrows). Additionally, ELA and Apelin are expressed in syncytiotrophoblasts (black arrows). No staining was observed in the rabbit IgG control staining. Antibody-specific DAB staining is shown in brown, haematoxilin counterstain was used to stain nuclei blue. (**b)** In HTR8/SVneo cells ELA, APLNR and Apelin are weakly expressed in adherent proliferating cells, while ELA expression becomes strong in invaded cells. No staining was observed in the rabbit IgG control staining. (**c)** Invasion assays show that increasing levels of ELA increase the number of invaded cells. (**d)** Blocking APLNR using non-peptide antagonist ML221 does not significantly reduce the effect of ELA on invasion, while ML221 itself appears to increase invasion although not significantly. (**e)** siRNAs targeting *APLNR* were tested in HTR8/SVneo cells that were stably transfected with a vector expressing *APLNR*. siRNA APLNR-3 and APLNR-4 were directed against untranslated regions not present in the vector and did not induce knockdown of *APLNR* mRNA. APLNR-1 and APLNR-2 did show knockdown of *APLNR* of around 80%, and were used in further experiments. (**f)** APLNR protein knockdown was confirmed by making use of the NanoLuc luciferase fused to APLNR in the stable transfected vector performing luciferase assays at 48, 72 and 96 hours after transfection, showing clear knockdown at 96 hours. (**g)** Both ELA and Apelin induce invasion of HTR8/SVneo cells, but siRNA-mediated knockdown of *APLNR* does not significantly reduce the effect of ELA or Apelin on invasion. Transwell invasion assay experiments were repeated 3–5 times using 3–5 replicates per treatment. Data are presented as mean ± SEM and tested with one-way ANOVA followed by Bonferroni multiple comparisons test or with Student’s t-test in case of two data sets. *Indicates p < 0.05; **Indicates p < 0.01.

In first trimester human placenta ELA and Apelin are expressed in villous cytotrophoblasts, syncytiotrophoblasts and in distal column extravillous trophoblasts, while their putative receptor APLNR is expressed in villous cytotrophoblasts and distal column extravillous trophoblasts (Fig. 1a). HTR8/SVneo cells, a first trimester extravillous trophoblast cell line immortalized with simian virus 40 large T antigen (SV40), shows a similar extravillous trophoblast expression pattern with very weak expression in proliferating adherent cells (Fig. 1b). These overall weak protein expression levels are consistent with a published GEO Dataset of HTR8/SVneo RNA-sequencing results (GSE105783) providing no counts for both ELA and APLNR, while Apelin with a mean of 27 counts fell within the lower quartile of expressed genes. However, upon invasion the expression of endogenous ELA is highly increased, but no evident expression was seen for Apelin and APLNR (Fig. 1b). As endogenous expression of ELA appears to be low or absent in proliferating proximal extravillous trophoblasts, effects of ELA on invasion can only be studied by addition of exogenous recombinant ELA peptide. Previous data obtained in JAR choriocarcinoma cells indicated ELA was able to increase the invasion capacity of these cells^13^. We therefore first tested if exogenous ELA was also able to increase the invasion of HTR8/SVneo cells. Increasing the amount of recombinant ELA peptide indeed increased the number of invaded cells in transwell invasion assays (Fig. 1c). To investigate if ELA induced invasion through APLNR we used ML221, a non-peptide antagonist of APLNR. Although a small decrease in invasion was observed upon treatment of the cells with both ELA and ML221 compared to treatment with ELA only, this was not significant, while a non-significant increase was observed when the cells were treated with ML221 only (Fig. 1d). These results suggested that the invasion effect of ELA, at least in part, is not achieved through signaling via APLNR, while blocking APLNR by ML221 appears to directly affect levels of other ligands involved in invasion. As ML221 treatment appears to affect signaling of other ligands involved in invasion, it was decided to repeat the transwell invasion assays in combination with siRNA-mediated knockdown of APLNR. To confirm the efficiency of the knockdown by quantitative PCR and luciferase assays, we used HTR8/SVneo cells that were stably transfected with a vector expressing *APLNR* fused to NanoLuc luciferase, as endogenous expression of *APLNR* mRNA was barely detectable making knockdown efficiency difficult to estimate. Two of the four siRNAs that were tested, those which had target locations within the cDNA of *APLNR* and therefore present in the stably transfected cells, showed a knockdown of approximately 80% (Fig. 1e). These siRNAs were used to confirm knockdown at protein level by performing luciferase assays (Fig. 1f), and were used in subsequent invasion assays. Invasion assays were performed with both recombinant ELA and Apelin in combination with *APLNR* knockdown. Both ELA and Apelin were able to significantly increase invasion while knockdown of *APLNR* did not yield a significant reduction of invasion (Fig. 1g), indicating that both ELA and Apelin are able to induce differentiation of HTR8/SVneo cells, but that these effects are, at least in part, independent of signaling through APLNR.

### In first trimester placental explants ELA affects outgrowth morphology and trophoblast proliferation

**Figure 2:**
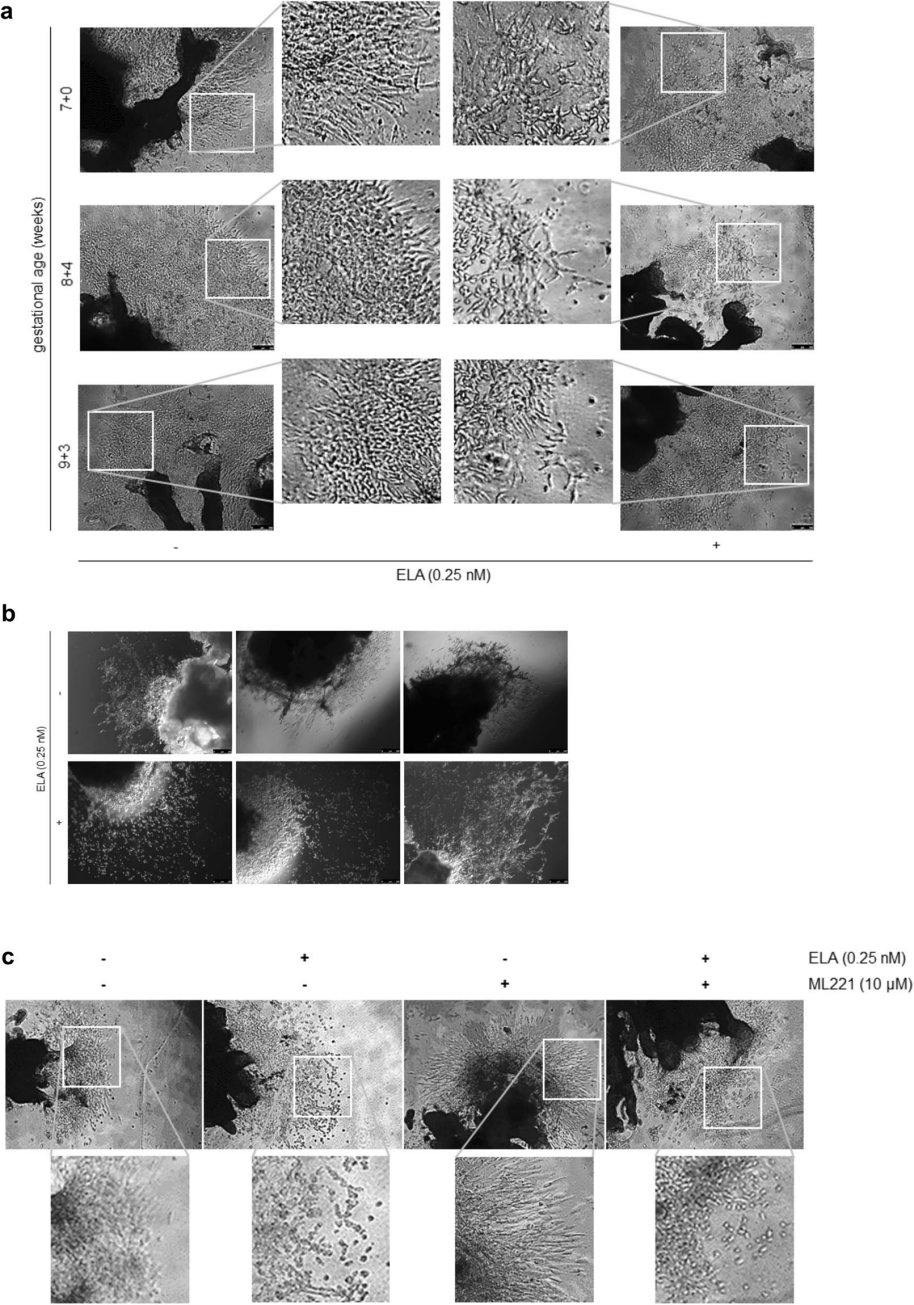
ELA affects first trimester placental explant outgrowth morphology. (**a**) Representative images of first trimester placental explants with and without addition of ELA. Exogenous ELA added to explants changes their morphology with the outgrowth more diffuse and less organized. n = 22 placentas, 5 explants per treatment. (**b**) Similar as **a**, but to enhance image contrast media was removed prior to taking pictures. These explants could therefore not be used in downstream experiments. (**c**) Representative images of explants treated with both ELA and APLNR non-peptide antagonist ML221. Addition of ML221 does not return outgrowth morphology to control appearance. ML221 alone shows no effect on outgrowth morphology. n = 12 placentas, 5 explants per treatment.

**Figure 3:**
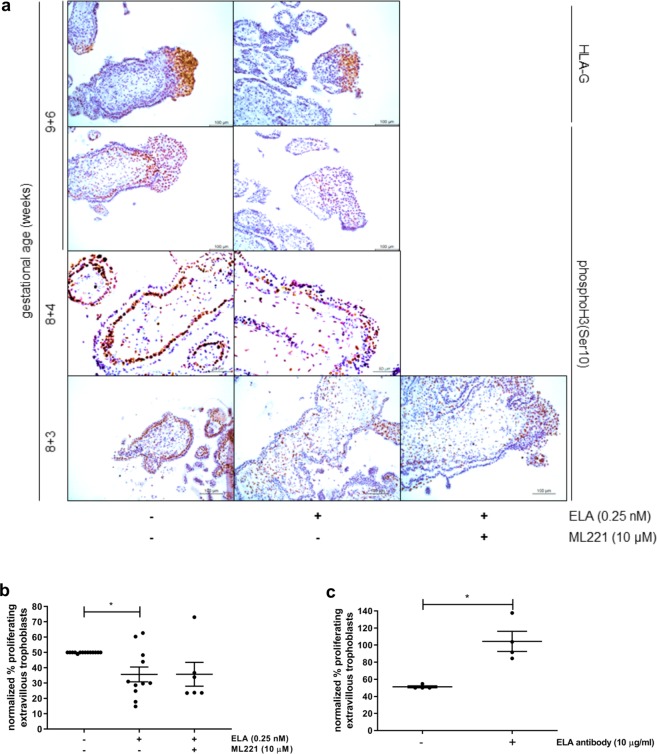
ELA affects first trimester placental explant trophoblast proliferation. (**a)** HLA-G immunostaining (brown color top panel) indicates the location of extravillous trophoblasts. PhosphoH3(Ser10) immunostaining (brown color lower 3 panels) identifies proliferating cells undergoing mitosis. In extravillous trophoblasts of explants treated with ELA proliferation appears to decrease. In control explants almost all villous cytotrophoblasts proliferate, while this is much less apparent in explants treated with ELA. Treatment with ELA in combination with ML221 does not return villous cytotrophoblast proliferation to the level as seen in control explants. (**b)** Quantifying the percentage of proliferating extravillous trophoblasts observed by immunohistochemistry shows a significant decrease upon treatment with ELA which cannot be rescued by the addition of ML221. P = 0.08 between controls and explants treated with ELA and ML221. n = 12 placentas, 5 explants per treatment. Bars represent mean ± SEM and data was tested with one-way ANOVA followed by Bonferroni multiple comparisons test. *Indicates p < 0.05. (**c**) Treatment of explants with an ELA neutralizing antibody shows a significant increase of extravillous trophoblast proliferation upon quantifying immunohistochemistry stainings. n = 4 placentas, 5 explants per treatment. Bars represent mean ± SEM and data was tested with a Student’s t-test. *Indicates p < 0.05.

Next, we were interested in the behavior of recombinant ELA in an *ex vivo* model system, i.e. first trimester placental explants. Placental explants were treated with ELA hypothesizing we would observe a significant increase in extravillous trophoblast outgrowth. Although no clear differences were seen in the extent of outgrowth, we did consistently see differences in outgrowth morphology. Where control outgrowth shows compact and organized outgrowth structures, ELA treated outgrowths were more diffuse and less organized, suggesting differentiation to a more advanced, i.e. invasive, phenotype (Fig. 2a,b, Supplementary Fig. 1). Although immunohistochemistry on first trimester placenta tissue showed APLNR expression in villous cytotrophoblasts and distal column extravillous trophoblasts (Fig. 1a), treatment of ELA in combination with APLNR antagonist ML221 in placental explants also did not reverse the effect ELA has on the morphology of the outgrowths, while ML221 on its own showed no effect (Fig. 2c). Next, immunohistochemistry was performed on fixed explants treated with ELA alone or in combination with ML221 (Fig. 3a). Extravillous trophoblasts were identified by staining with HLA-G antibody. In parallel sections we also stained for phosphoH3(Ser10) which marks proliferating cells to identify cells undergoing mitosis. We next quantified the percentage of proliferating extravillous trophoblasts, normalized the results obtained in ELA treated explants to the results obtained in control explants within each placenta, and found a significant decrease in proliferating extravillous trophoblasts in explants treated with ELA (Fig. 3b). This effect again could not be reversed to control levels by ML221 treatment. Next to changes in extravillous trophoblast proliferation we also observed changes in villous cytotrophoblast proliferation (Fig. 3a); control explants show a continuous row of proliferating villous cytotrophoblasts, while this row is intermittent in explants treated with ELA alone or in combination with ML221. Explants were also treated with an antibody raised against ELA to neutralize its effects^11^. We did not observe changes in outgrowth morphology upon this treatment, but quantifying proliferating extravillous trophoblasts after immunohistochemical staining did show a significant increase in the percentage proliferating cells (Fig. 3c). These data suggest that in an *ex vivo* placenta model ELA affects differentiation of extravillous trophoblasts leading to changes in outgrowth morphology and trophoblast proliferation, which again appear to be, at least partially, independent of APLNR.

### Circulating ELA levels are lower in first trimester plasma of women with a healthy BMI later developing preeclampsia

**Figure 4:**
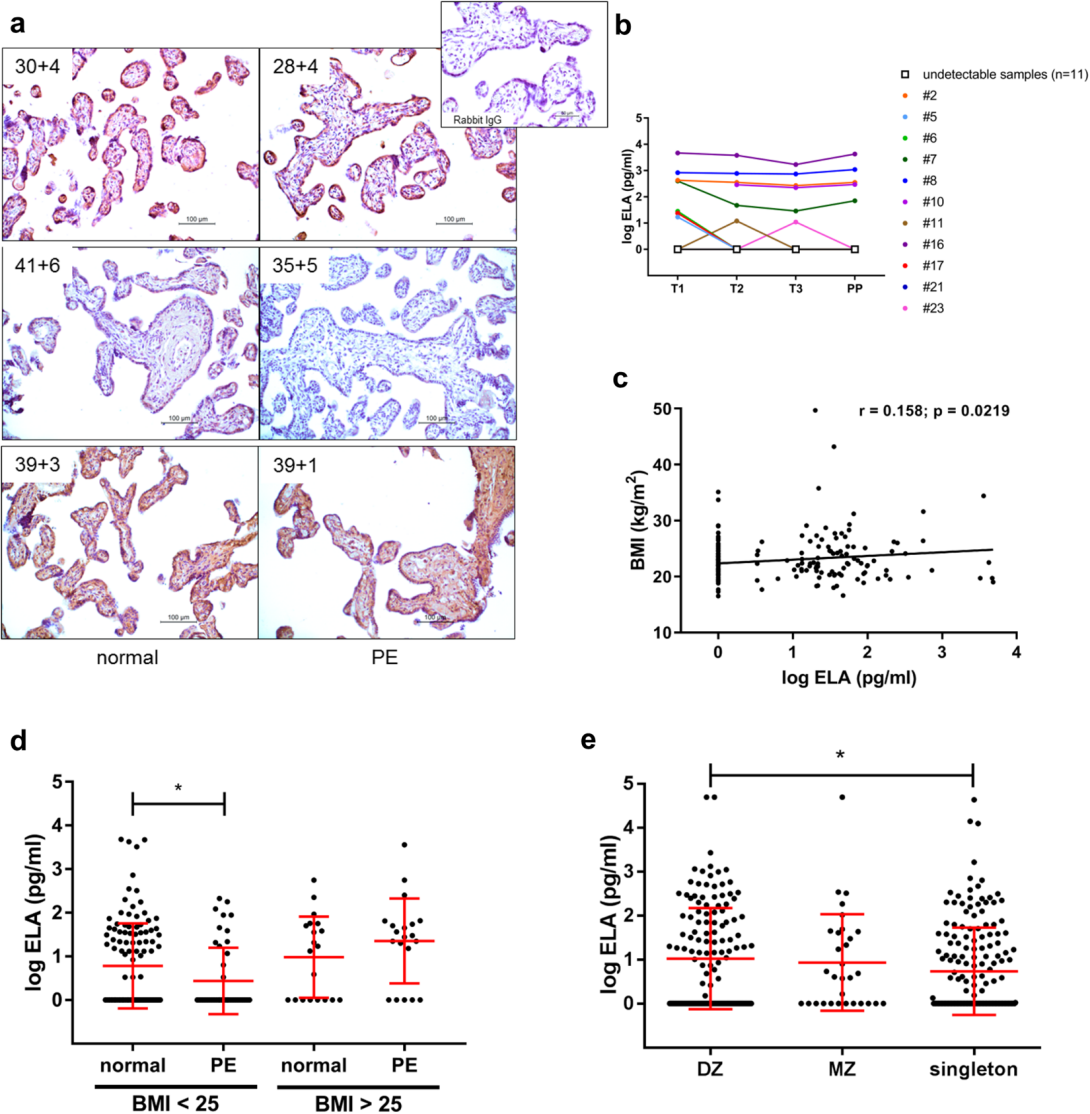
Circulating ELA levels are highly variable between women and are lower in first trimester plasma of women with a healthy BMI later developing preeclampsia. (**a)** Immunohistochemical staining of (pre)term placentas of normal and preeclamptic pregnancies does not show consistent differences in ELA expression levels. No staining was observed in the rabbit IgG control staining. (**b)** Serum samples from the longitudinal RADAR cohort (n = 22) shows ELA levels are highly variable between women and below detection limit (1 pg/ml) throughout pregnancy and postpartum in half of the women. (**c)** The plasma samples from the ABCD cohort (n = 139 normal and n = 66 preeclamptic pregnancies) show a weak but significant correlation between BMI and first trimester circulating ELA levels. (**d)** Stratifying the ABCD cohort in healthy BMI (<25) and BMI > 25 shows significantly lower ELA levels in women with a healthy BMI later developing preeclampsia compared to normal pregnancies. (**e)** Serum samples from the twin study cohort show that in third trimester ELA levels are significantly higher in dizygotic (DZ) twin pregnancies (n = 189) compared to singleton pregnancies (n = 251). MZ indicates monozygotic (n = 42). Data are presented as mean ± SD and tested with one-way ANOVA followed by Bonferroni multiple comparisons test. *Indicates p < 0.05.

We first performed immunohistochemistry using ELA antibody on (pre)term placental tissues of normal and preeclamptic pregnancies, but were unable to detect consistent downregulation of ELA in preeclamptic samples (Fig. 4a). Interestingly, in some of the term placental samples in addition to trophoblasts also stromal cells showed strong ELA staining.

We next decided to measure circulating ELA in three different cohorts. The Resistance of Aspirin During and After pRegnancy (RADAR) cohort contains longitudinal serum samples of pregnant women at high risk to develop placenta mediated complications based on previous pregnancies (Table 1). In 50% of the women in this cohort ELA could not be detected throughout pregnancy and postpartum (Fig. 4b). Furthermore, the ELA levels were highly variable between women and did not change dramatically during pregnancy or postpartum, although a slightly higher level is visible in the first trimester.

**Table 1:** Demographic and clinical characteristics of the RADAR cohort.

N	Normal	Complicated	P value
	13	9	
Maternal age (years)	33.2 ± 4.3	33.1 ± 3.4	0.95
Pre-pregnancy BMI (kg/m^2^)	23.4 ± 6.6	24.8 ± 4.4	0.61
1st trimester sampling (weeks)	13.0 ± 1.6	12.1 ± 2.2	0.28
2nd trimester sampling (weeks)	21.1 ± 0.9	21.8 ± 1.5	0.23
3rd trimester sampling (weeks)	30.6 ± 1.1	31.4 ± 0.8	0.09
Postpartum sampling (weeks)	16.6 ± 3.8	15.6 ± 2.4	0.49
Gestational age at delivery (weeks)	38.8 ± 0.9	37.6 ± 2.3	0.15
Birth weight (grams)	3229 ± 395	3044 ± 1022	0.62
PIH (n)	NA	5	
PE (n)	NA	3	
Birth weight percentile <p10 (n)	NA	3	

Values are mean ± SD. P values obtained from unpaired t-tests.

The second cohort we used was a subgroup of the Amsterdam Born Children and their Development (ABCD) study consisting of first trimester plasma samples (Table 2). All available samples from preeclamptic women were used together with randomly selected controls. This cohort showed a weak but significant correlation between BMI and circulating ELA levels (Fig. 4c). We therefore stratified the cohort in healthy BMI (<25) and BMI > 25 by which we eliminated the significant differences in BMI between controls and preeclamptic women within the stratified groups. Now, significantly lower levels of circulating ELA were found in women with a healthy BMI later developing preeclampsia compared to women with normal BMI and uncomplicated normal pregnancies (Fig. 4d). Here, early- and late-onset preeclampsia samples were analyzed together. Interestingly, when we looked at the two disease subsets separately, we found similar significance when comparing late-onset to control samples, while the early-onset did not show significance, potentially due to the small sample size. Similar BMI-dependent ELA level observations were made in first trimester RADAR samples developing a complicated pregnancy (Supplementary Fig. 5), but sample numbers are too low for statistical analysis.

**Table 2:** Demographic and clinical characteristics of the subgroup from the ABCD cohort.

N	Normal	PE	P value
	139	66	
Maternal age (years)	31.0 ± 4.9	31.0 ± 5.6	0.97
Pre-pregnancy BMI (kg/m^2^)	22.3 ± 3.4	24.0 ± 4.8	0.00
Gestational age at sampling (days)	94.2 ± 24.7	93.4 ± 25.5	0.84
Gestational age at delivery (days)	278.2 ± 14.9	270.2 ± 22.9	0.01
Birth weight (grams)	3510 ± 644	3062 ± 874	0.00
Early-onset (n)	NA	15	
Late-onset (n)	NA	51	

Values are mean ± SD. P values obtained from unpaired t-tests.

The third cohort used was part of the twin study (Table 3) from which we obtained second and third trimester serum samples. This cohort was included to study if a second placenta in twin pregnancies would lead to increased levels of circulating ELA secreted by these two placentas compared to singleton pregnancies. In third trimester samples a significant rise in ELA levels was found in samples obtained from dizygotic twin pregnancies compared to singleton pregnancies (Fig. 4e), while this rise was not yet visible in second trimester.

**Table 3:** Demographic and clinical characteristics of the twin study cohort.

N	Singleton	Monozygotic (MZ)	Dizygotic (DZ)	P value
	251	42	189	
Maternal age (years)	33.9 ± 4.1	32.0 ± 4.5	33.3 ± 3.8	0.01
BMI at 20 weeks (kg/m^2^)	25.1 ± 4.0	26.3 ± 3.4	25.9 ± 4.3	0.08
Gestational age at delivery (weeks)	39.5 ± 1.7	36.9 ± 1.7	37.0 ± 1.6	0.00
Birth weight (grams)	3424 ± 638	2655 ± 426	2651 ± 430	0.00

Values are mean ± SD. P values obtained by ANOVA.

These data combined indicate that circulating ELA levels are highly variable between women and are affected by BMI but are not strongly affected by pregnancy. By stratifying the samples obtained in first trimester in BMI < 25 and BMI > 25, a significant decrease in circulating ELA was observed in women with a healthy BMI later developing preeclampsia.

## Discussion

Here we show that ELA and Apelin in the first trimester extravillous trophoblast cell line HTR8/SVneo are able to increase the invasive capacities of these cells and that this is, at least in part, independent from signaling through the APLNR as shown by lack of effect of treatment with APLNR antagonist ML221 or transfection of siRNAs directed against *APLNR*. The independence from APLNR signaling was also seen in our *ex vivo* placental explants model system where changes in outgrowth morphology, suggesting differentiation into a more invasive phenotype, and percentage of proliferating extravillous trophoblasts changed upon treatment with ELA, while this could not be reversed by combined treatment with APLNR antagonist ML221. The option that ELA signals via an alternate receptor has been suggested to occur in both human embryonic stem cells and ovarian clear cell carcinoma cells^11,14^, and there is also evidence that Apelin may signal via an alternative receptor^15^. If this unidentified receptor is identical for the two peptides remains to be investigated. Interestingly, a study was recently published that also used ELA treatment in combination with ML221 in HTR8/SVneo cells^16^. Here they also find a comparable increase in invasion upon ELA treatment, i.e. threefold, but unlike our findings they do find a significant decrease upon combined treatment with ELA and ML221. In our treatments we used concentrations of ELA and Apelin that were at the higher end of the levels we measured to be secreted by first trimester placental explants after 4 to 5 days in culture (0.25 nM and 10 nM, respectively). In contrast, the study by Zhou *et al*. used a concentration of ELA that was 20 times higher (5 nM). It is plausible to assume that this extremely high concentration of ELA used in their study caused an upregulation of APLNR expression and signaling, which could subsequently be blocked by ML221.

In term placental tissues we could not detect clear differences in ELA protein expression between healthy and preeclamptic placentas, the potential decrease observed in preeclamptic tissues was seen in tissues with also a lower gestational age, while the healthy and preeclamptic tissue with matched gestational age (39 + 3 vs. 39 + 1 weeks) clearly did not show any difference in ELA expression. To be more conclusive more gestational age-matched samples should be used in a quantitative protein assay. Additionally to ELA expression in cells of trophoblast origin, in some of the term placenta samples we also observed strong staining in stromal cells. This indicates that trophoblasts are not the only source of placental ELA and stromal cells might also play a functional role in placental development and/or preeclampsia.

Circulating ELA levels measured with our custom sandwich ELISA were found to be extremely variable between women ranging from undetectable (less than 1 pg/ml) to 50,000 pg/ml, while levels within a woman during pregnancy and postpartum were relatively stable with only a potential small rise in first trimester. Furthermore, we found a significant correlation with BMI. This correlation might reflect the induced cardiovascular stress upon higher BMI levels leading to induction of ELA secretion, as ELA has been shown to have hypotensive and inotropic properties^17^. Additionally, we observed that women in third trimester carrying a dizygotic twin pregnancy have increased ELA levels compared to singleton pregnancies. This increase might reflect the additional secretion of ELA by the additional placenta, but most likely is also due to induced ELA secretion upon increased cardiovascular stress accompanying a twin pregnancy. All of these findings clearly suggest that the placenta is not the major source of circulating ELA in pregnancy, and that ELA expression levels in placental tissue cannot be directly translated to circulating levels of ELA. Because of the observed correlation with BMI we decided to stratify our cohorts in healthy BMI samples (<25) and samples with a BMI > 25. This demonstrated a significant decrease of ELA levels in first trimester plasma samples of women later developing preeclampsia, but only in the group with healthy BMI levels. We recently performed a systematic review with dataset quality assessment in which we discuss the studies performed so far on circulating ELA levels in healthy and preeclamptic pregnancies^18^. We concluded that for early-onset preeclampsia no differences in circulating levels could be found compared to healthy pregnancies. Regarding late-onset preeclampsia the two studies with sufficient quality scores showed opposite results; the study by Zhou *et al*. found decreased levels of ELA in late-onset preeclamptic patients compared to controls^16^, while the study by Panaitescu *et al*.^19^ found increased levels of ELA in late-onset preeclamptic women. Interestingly, the study by Zhou *et al*. included women with mean BMI levels below 25, while the study by Panaitescu *et al*. included women with a median BMI of around 28, which would stratify the majority of these women in the BMI > 25 group. Both of these studies therefore appear to be comparable to the results we found, i.e. a significant decrease in ELA levels in women with BMI < 25 while in our group with women with BMI > 25 we found a trend towards an increase of ELA in women later developing preeclampsia. An additional remark regarding previous studies measuring ELA by ELISA has to be made; as previous studies all used commercial ELISA kits, we have recently measured several samples with both our custom ELISA and a commercial kit recommending sample preparation by peptide extraction^20^. When performing peptide extraction the ELA levels obtained by the commercial kit mirrored the levels obtained by our custom ELISA, while when omitting sample preparation the ELA levels no longer showed large inter-individual variations. Both of the studies mentioned above are inconclusive to the fact if sample preparation was performed, which makes it difficult to be certain if these studies can be compared to the results of this study.

In the present study we show that ELA affects extravillous trophoblast differentiation as it is able to increase invasiveness of extravillous trophoblasts *in vitro*, is able to change outgrowth morphology and reduce trophoblast proliferation *ex vivo*, and that these effects are, at least in part, independent of signaling through the Apelin Receptor. Secondly, we show that circulating levels of ELA are highly variable between women, correlate with BMI, but appear to be significantly reduced in first trimester plasma of women with a healthy BMI later developing preeclampsia. These results indicate that the large variability and BMI dependence of ELA levels in circulation most likely make this peptide an unlikely candidate to function as a first trimester biomarker to screen for women at risk to develop preeclampsia. However, in the future first trimester administration of ELA or a derivative, possible once a first trimester preeclampsia biomarker would become available, might be considered as a potential preeclampsia treatment option as ELA is able to drive extravillous trophoblast differentiation.

## Methods

### Cell culture and transfection

HTR8/SVneo extravillous trophoblast cells were obtained from ATCC and grown in RPMI media supplemented with FBS, HEPES, sodium pyruvate, glucose and pen-strep at 37 °C, 5% CO_2_. In case of transfection experiments 150,000 HTR8/SVneo cells were transfected with 80 pmol *APLNR* siRNAs (Qiagen, Germany, Flexitube Genesolution, a package of 4 preselected siRNAs targeting *APLNR* catalogue # GS187) or non-targeting siRNAs (Qiagen) using 2.5 µl XtremeGENE HP transfection reagent (Sigma). 48 hours after transfection, the cells were harvested and stored at −80 °C or harvested to be used in invasion assays. To test the efficiency of siRNA-mediated knockdown of *APLNR*, stable transfected HTR8/SVneo cells were used that contained the pNLF1-secN Hygro vector (Promega) in which *APLNR* cDNA was cloned using PvuI and XbaI restriction sites, providing a protein fusion of NanoLuc luciferase with APLNR. siRNA-mediated APLNR protein knockdown was confirmed by performing Nano-Glo luciferase assays (Promega) according to the manufacturer’s instructions. To correct for the fact that in luciferase assays the cells remain proliferative while this is not the case in invasion assays, the RLU obtained in the luciferase assays were normalized to the percentage of cells with sufficient siRNA transfection levels to obtain knockdown, as determined by transfecting a fluorescently labelled siRNA (siGLO, Dharmacon) in parallel to the luciferase assays and measure its signal intensity over time by FACS.

### RNA isolation and quantitative PCR

RNA was isolated using the RNeasy Mini Kit (Qiagen) followed by cDNA synthesis using random hexamers and M-MLV reverse transcriptase (Promega). Quantitative PCRs were performed in triplicate on a Lightcycler 480 instrument using SYBR Green Supermix (Roche) according to the manufacturers protocol in combination with primers recognizing APLNR (CTGAGCGCCTCTTCTCCCGG; GCAACGGTCTGGTGCTCTGGA), and PSMD4^21^ (GGCAAGATCACCTTCTGCAC; CTTCCCACAAAGGCAATGAT) and YWHAZ^22^ (AATGGCTTCATCGAAAGCTG; CTGGCCCTCAACTTCTCTGT) as reference genes. The relative quantity (RQ) of gene expression was calculated using RQ = 2^−∆Ct^, where ∆Ct = Ct target gene − Ct geometric mean reference genes.

### Invasion assays

50,000 HTR8/SVneo cells were seeded on 100 µl 10x diluted Matrigel (Corning) coated 8.0 μm cell culture inserts in the absence or presence of ELA peptide (Tocris), Apelin-13 (Sigma) and ML221 non-peptide APLNR antagonist (Sigma). When invasion assays were performed using siRNA-transfected cells (as described above), from the harvested transfected cells 50,000 cells were seeded on 100 µl 16x diluted Matrigel (Corning) coated 8.0 μm cell culture inserts in the absence or presence of ELA peptide (Tocris) or Apelin-13 (Sigma). Invasion took place for 48 hours after which the membranes were fixed, mounted in Vectashield with DAPI (VectorLabs) and coverslipped, after which the cells on the underside of the membrane were counted. To count the cells pictures were taken of 9 random fields per membrane after which ImageJ was used to quantify the number of cells per picture.

### First trimester placental explants

First trimester placenta collections were approved by the AMC Biobank review committee, and were obtained at the time of elective terminations of pregnancy. Informed consent was obtained from each patient. Small fragments of placental villi from 5 to 12 weeks gestation were dissected from the placenta and placed in culture plate wells coated with collagen I (R&D systems). Placental villous explants were cultured for a maximum of 5 days at 5% O_2_ and 37 °C in serum free DMEM/F12 media supplemented with pen-strep. Images of the explants were taken daily to monitor changes in outgrowth. At day 4 or 5 explants were fixed in 4% PFA and processed for immunohistochemistry.

### Immunohistochemistry and immunofluorescence

Term placenta samples were collected through the Preeclampsia And Non-preeclampsia Database (PANDA^21^) an obstetrical biosample effort approved by the medical ethical committee of the Academic Medical Center. First trimester placenta samples were collected as described above. First and third trimester placenta tissue and first trimester placental explants were sectioned and subjected to standard immunohistochemistry procedures. Antigen retrieval was performed using microwave pre-treatment in sodium citrate buffer (antigen retrieval was omitted when using the ELABELA antibody). Blocking was done in 5% BSA, followed by overnight primary antibody incubations in 1% BSA at 4 °C. The following primary antibodies were used: ELABELA (custom by^11^), APLNR (Sigma, SAB2700205), Apelin (GeneTex, GTX37465), HLA-G (Novus Biologicals, NB500-302) and phosphoH3(Ser10) (Sigma, 09-797). Finally, Powervision Poly-HRP secondary antibodies were used followed by DAB staining and counterstaining using haematoxilin. ImageJ was used to count the number of proliferating extravillous trophoblasts. For immunofluorescence, HTR8/SVneo cells were fixed, followed by blocking in 3% BSA. Primary antibodies were added overnight at 4 °C. Finally, Alexafluor-488 labeled secondary antibodies were used followed by DAPI counterstain.

### Human serum and plasma samples

Human serum samples were obtained from the Amsterdam University Medical Centers, location VUmc (RADAR (longitudinal sampling in n = 13 normal and n = 9 complicated pregnancies) and twin study cohorts (second and third trimester sampling in n = 251 singleton, n = 42 monozygotic, and n = 189 dizygotic twin pregnancies)) and the plasma samples were obtained from the Amsterdam University Medical Centers, location AMC (ABCD cohort (first trimester sampling in n = 139 normal and n = 66 preeclamptic pregnancies)). The ABCD study was approved by the medical ethical committee of the Academic Medical Center. The RADAR cohort and twin study cohort were approved by the medical ethical committee of the VU University Medical Center. All studies were performed in accordance with relevant guidelines and regulations. Tables 1–3 show the demographic and clinical characteristics of the included women in all three cohorts. All women signed informed consent for use of material and clinical information for research purposes. Since the ABCD samples were collected in 2003, complications during pregnancy were defined according to the ISSHP 2001 guidelines in which preeclampsia is defined as *de novo* hypertension after 20 weeks gestation, returning to normal postpartum, and proteinuria. Early-onset preeclampsia was defined as occurring before 34 weeks gestation, late-onset occurring after 34 weeks gestation.

### ELISA

The custom ELA ELISA was performed as described previously^11^ with the following modifications: human plasma and serum samples were pretreated with 2.5 M urea for 10 minutes before applying the samples to the coated ELISA plate. Urea was added to release ELA from any binding proteins in the sample and did not interfere with the peptide-antibody interaction. Data on validation of the custom ELISA on human plasma and serum samples including the addition of urea can be found in Supplementary Figs. 2–4. ELA peptide used as a standard was diluted in pooled serum depleted from ELA by immune depletion using Immobilized Protein A resin slurry mixed with ELA antibody.

### Statistics

Statistical analyses were performed using Graphpad Prism version 8 software using unpaired t-tests in case of two groups or with one-way ANOVA (using the mean of technical replicates) followed by a Bonferroni multiple comparison test when comparing multiple groups, while cohorts consisting of serum or plasma samples were initially analyzed using SPSS version 25 software. To be able to include undetectable samples in data analysis we added 1 (pg/ml) to all ELA measurements before log transforming the data. Correlations were performed using bivariate Pearson correlation analysis. *P* values < 0.05 were considered significant.

## Supplementary information

Supplementary information
