# Supplementary material for: Peptide hormone ELABELA enhances extravillous trophoblast differentiation, but placenta is not the major source of circulating ELABELA in pregnancy
Source: Sci Rep. 2019 Dec 13;9:19077. doi: 10.1038/s41598-019-55650-5 (PMC6911039; doi:10.1038/s41598-019-55650-5)
Supplement: Supplementary file 1 — Supplementary information [file 41598_2019_55650_MOESM1_ESM.pdf]

Supplementary Information

Peptide hormone ELABELA enhances extravillous trophoblast differentiation, but placenta is not the major source of circulating ELABELA in pregnancy

Danai Georgiadou, Souad Boussata, Willemijn HM Ranzijn, Leah EA Root, Sanne Hillenius, Jeske M bij de Weg, Carolien NH Abheiden, Marjon A de Boer, Johanna IP de Vries, Tanja GM Vrijkotte, Cornelis B Lambalk, Esther AM Kuiper, Gijs B Afink, Marie van Dijk

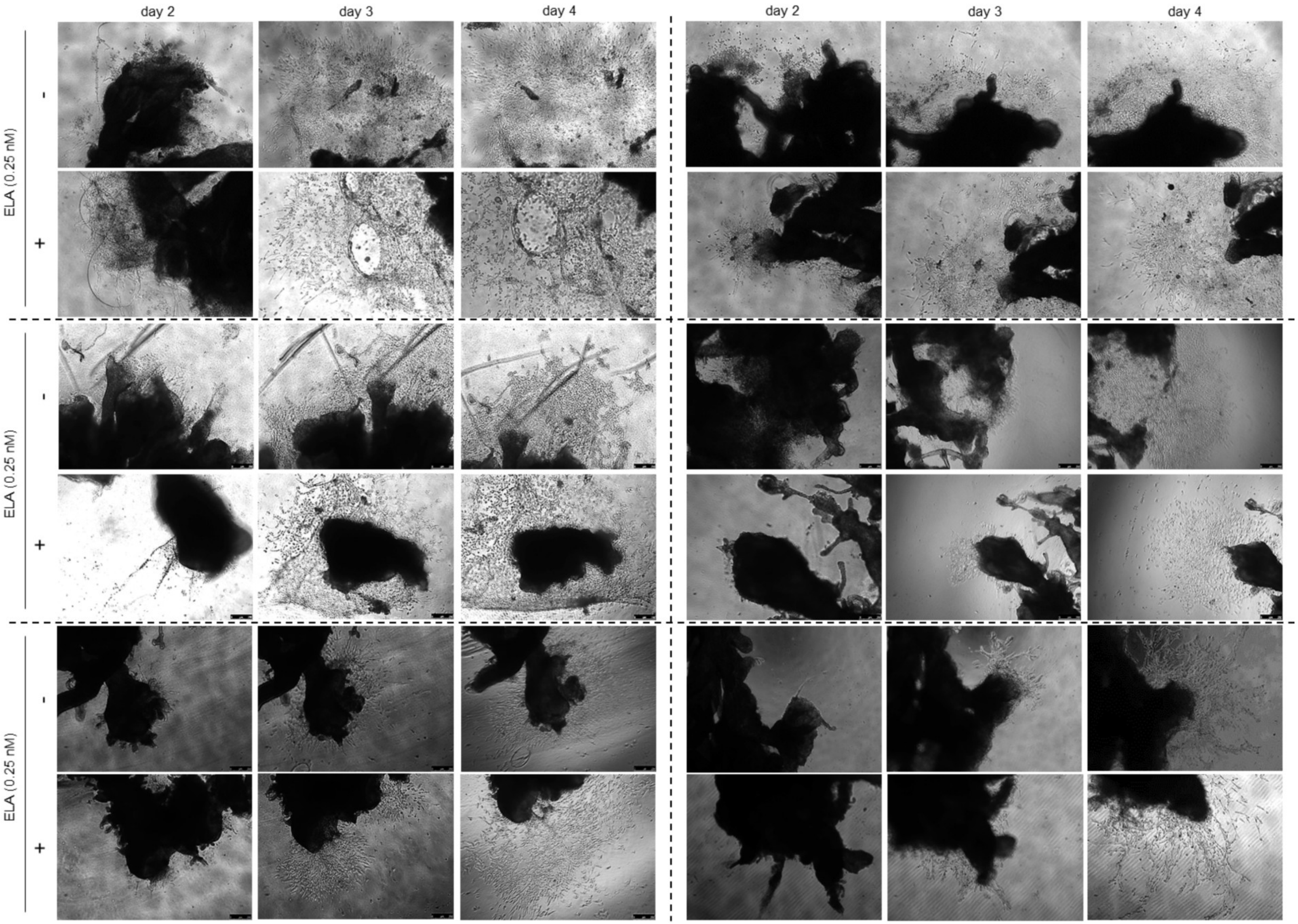

Supplementary Figure 1: Time course images of first trimester placental explants from six different placentas with and without addition of ELA. Exogenous ELA added to explants changes their morphology with the outgrowth more diffuse and less organized after 3 to 4 days in culture. Explant culture starts on day 1. On day 2 the first images are taken and recombinant ELA is added to the explants.

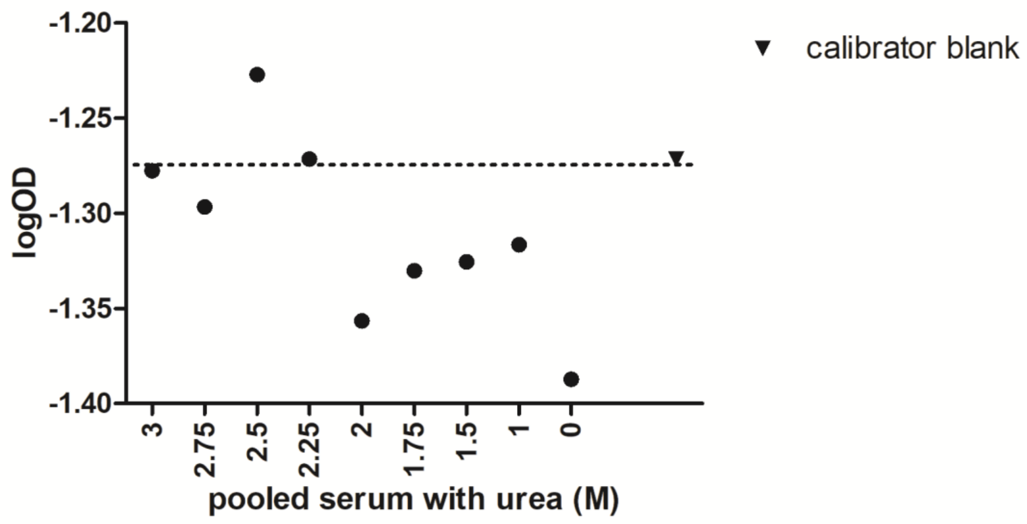

Supplementary Figure 2: Adding urea increases amount of detectable ELA.
Addition of increasing concentrations of urea to human serum increases the level of detectable ELA by the custom ELISA. A final concentration of 2.5M urea was found to be optimal.

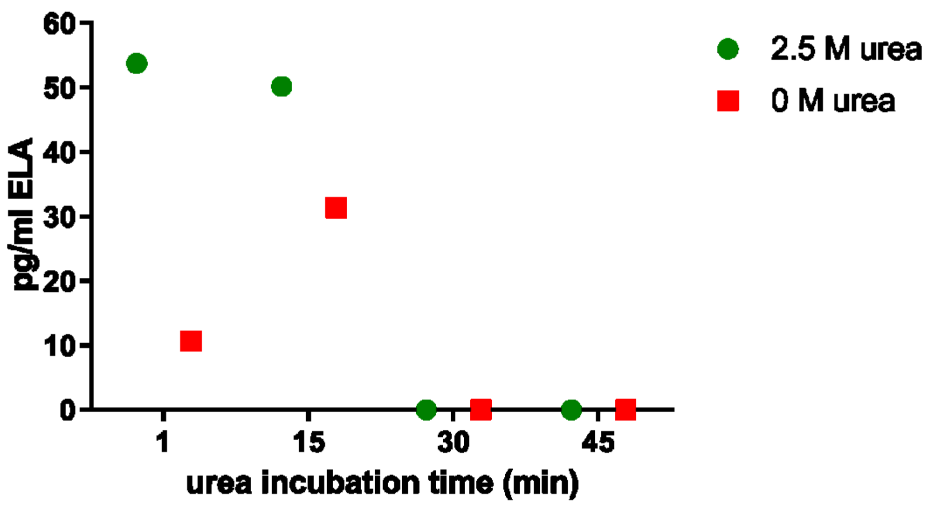

Supplementary Figure 3: Up to 15 minutes of sample treatment with 2.5M urea does not affect the ELA ELISA signal.
As the ELA peptide has a short half life, which might decrease even further upon urea treatment, the maximum amount of time was determined after which ELA peptide by custom ELISA could no longer be detected. At 30 and 45 minutes in both samples treated with and without urea ELA could no longer be detected, while at 1 and 15 minutes both samples could be detected, of which the urea treated sample showed higher ELA levels. We concluded that 10 minutes of urea incubation before adding the samples to the ELISA plate is optimal.

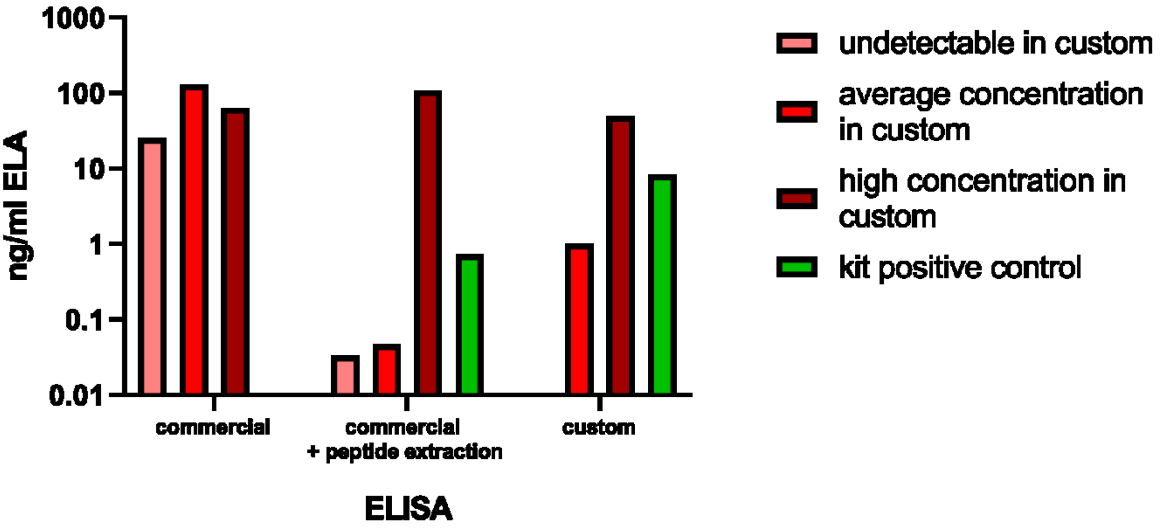

Supplementary Figure 4: Comparison of custom ELISA with commercial ELA ELISA kit with and without peptide extraction.

Three different pools of serum samples were used for this comparison representing samples that are undetectable in the custom ELISA, are detected at an average concentration in the custom ELISA, and are giving ELA levels rising above the calibrator curve in the custom ELISA. All currently available commercial ELA ELISA kits recommend sample preparation by peptide extraction; when peptide extraction is performed, the levels found by the custom ELISA and the commercial kit by Phoenix Pharmaceuticals are showing the same pattern including high inter-individual variation. When sample preparation is not performed, variation is much less and concentrations are between 10 and 100 ng/ml. Note that the commercial and custom ELISA calibrators differ by a 10-fold concentration (clearly visible in the kit positive control and average concentration sample), but it is unclear which of the calibrators is over- or under-diluted. Secondly, the custom ELISA measures up to 50 ng/ml which is why the high concentration sample is set at this concentration, as its signal went above the calibrator curve.

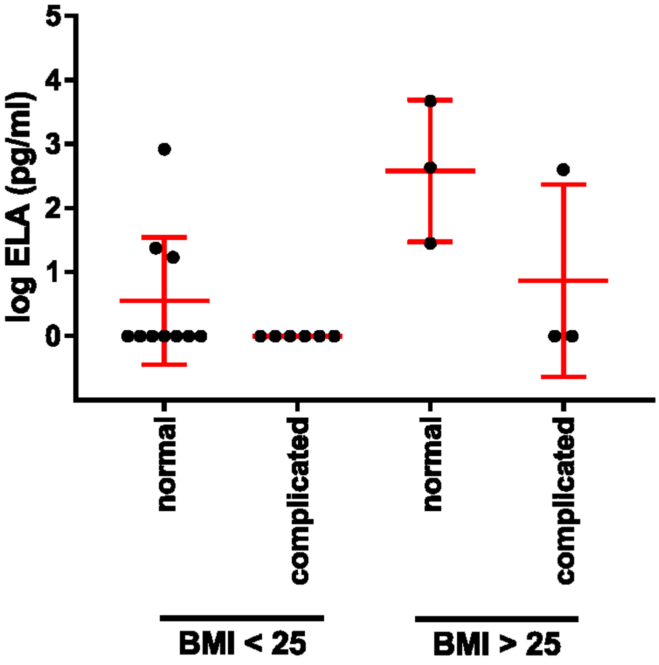

Supplementary Figure 5: The RADAR cohort shows a similar pattern as the ABCD cohort. Stratifying the RADAR cohort in healthy BMI (<25) and BMI >25 shows a similar pattern as the ABCD cohort with lower ELA levels in women with a healthy BMI later developing a complicated pregnancy compared to normal pregnancies.
